# Concomitant Surgical Ablation in Atrial Fibrillation Patients Undergoing Cardiac Surgery for Isolated Coronary and Aortic Valve Disease: A Multicentre Study from The Netherlands Heart Registration

**DOI:** 10.1093/ejcts/ezaf244

**Published:** 2025-07-28

**Authors:** M Agustina Bayón, Miriam A Scheurwater, Niels J Verberkmoes, Massimo A Mariani, Maaike M Roefs, Job van der Palen, Lukas R C Dekker, Yuri Blaauw, Thomas J van Brakel, Cardiothoracic Surgery Registration Committee of the Netherlands Heart Registration, S Bramer, R A F de Lind van Wijngaarden, B M J A Koene, J A Bekkers, G J F Hoohenkerk, A L P Markou, A de Weger, P Segers, D Stecher, R G H Speekenbrink, V G Hindori, W W L Li, E J Daeter, M M Mokhles, Y Douglas

**Affiliations:** Department of Cardiothoracic Surgery, University Medical Centre Groningen, Groningen 9713GZ, The Netherlands; Department of Cardiothoracic Surgery, Catharina Ziekenhuis, Eindhoven 5623EJ, The Netherlands; Department of Cardiothoracic Surgery, Catharina Ziekenhuis, Eindhoven 5623EJ, The Netherlands; Department of Cardiothoracic Surgery, University Medical Centre Groningen, Groningen 9713GZ, The Netherlands; Netherlands Heart Registration, Utrecht, The Netherlands; Department of Epidemiology, Medisch Spectrum Twente and Section Cognition, Data and Education, Faculty of Behavioural, Management and Social Sciences, University of Twente, Enschede 7522NB, The Netherlands; Department of Cardiothoracic Surgery, Catharina Ziekenhuis, Eindhoven 5623EJ, The Netherlands; Department of Cardiothoracic Surgery, University Medical Centre Groningen, Groningen 9713GZ, The Netherlands; Department of Cardiothoracic Surgery, Catharina Ziekenhuis, Eindhoven 5623EJ, The Netherlands

**Keywords:** coronary artery bypass grafting, aortic valve replacement, concomitant surgical ablation, atrial fibrillation

## Abstract

**Objectives:**

Concomitant surgical ablation (CSA) is recommended for atrial fibrillation (AF) patients undergoing cardiac surgery; however, its effects in non-mitral valve surgeries, specifically coronary artery bypass grafting (CABG) and aortic valve replacement (AVR), are less studied. This study aims to analyse outcomes and trends of CSA performance in the Netherlands.

**Methods:**

This nationwide multicentre study utilized data from the Netherlands Heart Registration. AF patients undergoing CABG or AVR between 2013 and 2021 were included. Temporal trends in CSA performance were analysed and a multivariable regression model adjusted for confounders when comparing CSA and non-CSA.

**Results:**

A total of 3260 patients were included, of which 1081 underwent CSA. CSA patients showed longer cardiopulmonary bypass (CPB) (111 vs 80, mean difference between groups: 31 min [95% CI, 27-34, *P* < 0.001]) and aortic cross clamping (AoX) times (67 vs 52, mean difference: 15 min [95% CI, 13-17, *P* < 0.001]). After correcting for confounders, CSA patients presented mean CPB and AoX times of 18 (95% CI, 16-21, *P* < 0.001) and 8 (95% CI, 6-10, *P* < 0.001) min longer. The CSA group showed higher survival rates (92.5% vs 86.4%, *P* = 0.039) and greater improvements in mental quality of life (QoL) (*P* = 0.047). CSA performance during CABG and AVR has increased significantly, from 29.7% in 2018 to 44.4% in 2021.

**Conclusions:**

CSA resulted in slightly longer CPB and AoX times but no significant differences in major complications. Regression analysis showed better survival rates and improved mental QoL for CSA. CSA performance in CABG and AVR has increased in the Netherlands.

## Graphical abstract

**Figure:**
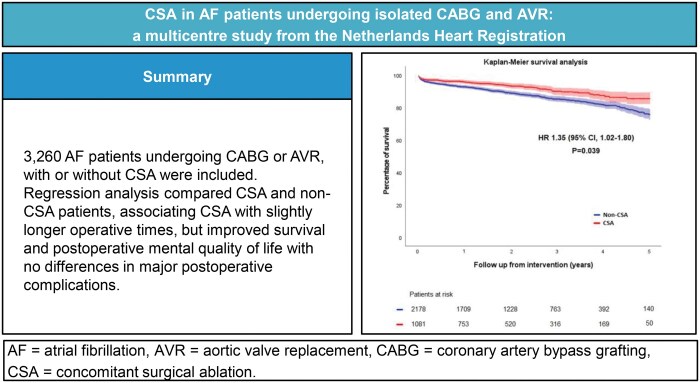

## INTRODUCTION

Atrial fibrillation (AF) is the most common cardiac arrhythmia, associated with significant morbidity, mortality, and increased healthcare costs.^1^^,^^2^ For AF patients undergoing cardiac surgery, concomitant surgical ablation (CSA) is recommended, due to its association with more favourable outcomes. The 2024 ESC/ACTS guidelines and the 2023 ACC/AHA guideline for the diagnosis and management of atrial fibrillation^3^^,^^4^ recommend CSA for mitral valve surgery (class I, evidence level A) and non-mitral valve surgery (class IIa, evidence level B). However, CSA is not consistently performed in patients with AF.^5^

Previous research demonstrated that CSA can be performed safely^6–9^ and is associated with positive postoperative outcomes including maintenance of sinus rhythm, reduced stroke incidence and improved survival.^6^^,^^7^^,^^9–11^

Most studies, including those reflected in recent guidelines,^3^^,^^4^ have primarily focused on outcomes of CSA in patients undergoing mitral valve interventions, where AF is frequent and its pathophysiology and aetiology with mitral valve disease often overlap. In contrast, the outcomes of CSA in combination with non-mitral valve surgery, particularly coronary artery bypass grafting (CABG) or aortic valve replacement (AVR), remain unexplored.

This study aims to evaluate CSA’s performance, outcomes, safety, and trends in AF patients undergoing CABG and AVR, the most commonly performed non-mitral valve surgeries.

## METHODS

### Source of study data

Data from all patients undergoing cardiac surgery were obtained from the Netherlands Heart Registration (NHR), a prospective, nationwide, mandatory quality-control registry dedicated to collecting comprehensive data on cardiovascular diseases and (surgical) interventions in the Netherlands.^12^^,^^13^ The NHR collects data including demographics, intervention type, parameters concerning perioperative morbidity and mortality, and mid-term survival from all cardiac surgeries performed. To obtain reliable data, the NHR has an advanced, certified data quality control system in place to ensure data completeness and quality.^14^

All patient, surgeon, and centre data were anonymized. No problems were reported regarding the quality of the long-term follow-up data from the NHR.

### Study design and population

The current study is a retrospective, multicentre cohort study of prospectively collected data from 16 cardiothoracic centres in the Netherlands. Included patients were aged 18 years and older, with a history of AF and undergoing isolated CABG or isolated AVR, with or without CSA, between 2013 and 2021. Exclusion criteria included: previous cardiac surgery, robotic or minimally invasive surgery, and other concomitant interventions. Follow-up closed in December 2021.

### Study end-points

Primary end-points were (in-hospital) mortality, cerebrovascular accident (CVA) or transient ischaemic attack (TIA), and cumulative survival. Additionally, trends in CSA performance over time in the Netherlands were analysed.

Mortality data were obtained from municipal records and were complete for all patients.

Secondary end-points included length of hospital stay, cardiopulmonary bypass (CPB), and aortic cross clamping (AoX) times. Additional end-points included pneumonia, urinary tract infection, re-admission to the intensive care unit (ICU), and postoperative renal failure. Other end-points included quality of life (QoL) comparison at baseline and 1-year follow-up, from SF-12 or SF-36 questionnaires.

### Missing values

The overall proportion of missing data was low (1.31%). All mandatory NHR variables were complete, but the EuroSCORE II had higher missing rates (9.0%).

### Statistical analysis

Normality of continuous variables was assessed by skewness and kurtosis, and by visual inspection of histograms. Continuous data were presented as mean with standard deviation (SD), or median with interquartile range (IQR) for non-normally distributed data. Group comparisons were conducted using the Student’s t-test or Mann-Whitney *U*-test for continuous variables and the Chi-squared or Fisher’s exact test for categorical variables.

A multivariable Cox regression model was implemented to evaluate time to event outcomes and mortality between patients undergoing CSA or not, while adjusting for statistically significant confounders. The model corrected for age, CPB use, renal impairment, LVEF <30%, peripheral vascular disease (PVD), diabetes mellitus (DM), and EuroSCORE II. All variables contributed meaningfully to the model. Cumulative survival was analysed with Kaplan-Meier curves, with differences between groups assessed by the log-rank test. Hazard ratios (HR) with 95% confidence intervals (CI) were obtained. CABG and AVR were combined into one mixed group to increase the sample size. An interaction analysis was conducted to exclude that the type of intervention (CABG or AVR) would modify the effect of performing CSA or not.

Binary logistic regression based on the abovementioned model was applied to analyse categorical outcomes. Linear regression was used for analysing continuous outcomes when the data were normally distributed. For skewed data, a log-transformation was applied to obtain normality of the data. Nevertheless, for interpretation purposes, the non-transformed means (SD) are presented.

Mixed linear models (MLMs) were used to evaluate pre- and postoperative QoL scores with the main parameter of interest being the group (CSA, non-CSA) by time interaction. A subanalysis compared survival and QoL outcomes between CABG and AVR patients.

A *P*-value < 0.05 was considered statistically significant. Analyses were performed using IBM SPSS Statistics 28 (SPSS Inc., Chicago, IL, USA) and RStudio 2023.12.1, Build 402.

### Ethical statement

The study was approved by the institutional review board MEC-U (W19.270) and conducted in agreement with the principles of the Declaration of Helsinki. A waiver for informed consent for analysis with the data of the NHR data registry was obtained.

## RESULTS

### Baseline characteristics

The NHR database contained 3260 AF patients who underwent either CABG (*N* = 2473) or AVR (*N* = 787) between 2013 and 2021. Of these patients, 1081 (33.16%) received CSA. Patients undergoing AVR were more likely to undergo CSA than patients undergoing CABG (30.9% vs 20.8%, *P* < 0.001). Patients undergoing CSA were younger (71 [65-75] vs 73 [67-77] years old, *P* < 0.001) and had a higher BMI (27.8 [25.2-30.6] vs 27.4 [24.8-30.4], *P* = 0.024) and a higher EuroSCORE II (2.28 [1.6-3.62] vs 1.91 [1.27-3.19], *P* < 0.001). They were also less likely to have a history of renal impairment (13.2% vs 18.1%, *P* < 0.001), PVD(10.5% vs 13.9%, *P* = 0.005), and DM (26.0% vs 29.4%, *P* = 0.041). Baseline characteristics are summarized in **Table 1**.

**Table 1.** Baseline Characteristics

	Non-CSA group N = 2179	CSA group N = 1081	*P*-value
AVR, *n* (%)	453 (20.79)	334 (30.90)	<0.001
CABG, *n* (%)	1726 (79.21)	747 (69.10)	<0.001
Sex (males), *n* (%)	1718 (78.8)	873 (80.8)	0.219
Age (years), median [IQR]	73 [67-77]	71 [65-75]	<0.001
BMI, median [IQR]	27.4 [24.8-30.4]	27.8 [25.2-30.6]	0.024
eGFR <50, *n* (%)	394 (18.1)	142 (13.2)	<0.001
DM, *n* (%)	642 (29.4)	281 (26.0)	0.041
LVEF <30%, *n* (%)	161 (7.4)	62 (5.7)	0.078
PAH >40 mmHg, *n* (%)	29 (1.3%)	8 (0.7%)	0.134
Chronic lung disease, *n* (%)	277 (12.7)	126 (11.7)	0.432
PVD, *n* (%)	302 (13.9)	113 (10.5)	0.005
CVA, *n* (%)	220 (10.1)	110 (10.2)	0.944
EuroSCORE II, median [IQR]	1.91 [1.27-3.19]	2.28 [1.6-3.62]	<0.001
Use of CPB, *n* (%)	1893 (86.9)	1033 (95.6)	<0.001

Abbreviations: AVR: aortic valve replacement; BMI: body mass index; CABG: coronary artery bypass grafting; CPB: cardiopulmonary bypass; CSA: concomitant surgical ablation; CVA: cerebrovascular accident; DM: diabetes mellitus; eGFR: estimated glomerular filtration rate; LVEF: left ventricular ejection fraction; PAH: pulmonary arterial hypertension; PVD: peripheral vascular disease.

### Regression analyses

**Figure 1.**
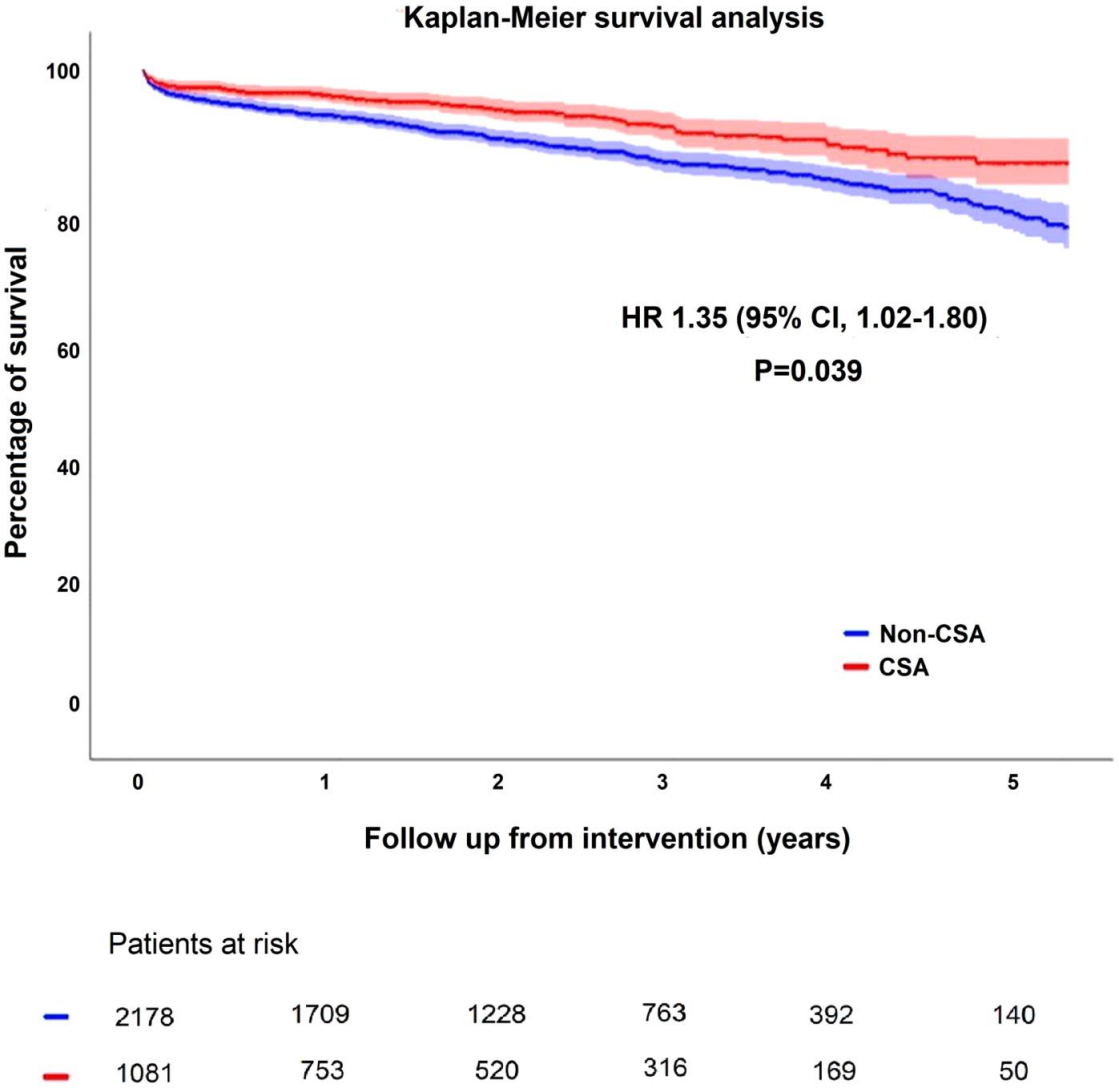
Kaplan-Meier Survival Analysis. Adjusted *P*-value for age, CPB use, renal impairment, LVEF <30%, PVD, DM, and EuroSCORE II. Abbreviation: CSA = concomitant surgical ablation

Mortality data were complete for all patients. A survival analysis was conducted (**Figure 1**; **Supplementary Table S2**), which showed higher cumulative survival rates in the CSA group over the follow-up period. The median follow-up time for the CSA group was 1.9 years (0.8-3.4) with a survival rate of 92.5%. For non-CSA patients, the median follow-up was 2.3 years (1.2-3.6) with a survival rate of 86.4%, HR 1.66 (95% CI, 1.30-2.13, *P* < 0.001).

A multivariable Cox regression model adjusted for the following significant confounders: age, CPB use, renal impairment, LVEF <30%, PVD, DM, and EuroSCORE II. An interaction term between the type of intervention (CABG or AVR) and the performance of CSA or not was tested to evaluate potential effect modification. This interaction was not statistically significant (*P* = 0.267) and did not improve model fit; therefore, it was excluded from the analysis.

After adjustment for confounders, the observations in the survival analysis became subtler but remained statistically significant, HR 1.35 (95% CI, 1.02-1.80, *P* = 0.039).

Linear regression analysis revealed longer CPB times in the CSA patients (111 vs 80 min with a difference of 31 min between groups [95% CI, 27-34, *P* < 0.001]). AoX times were also longer in the CSA group (67 vs 52 min, with a difference of 15 min [95% CI, 13-17, *P* < 0.001]). After correcting for confounders, the delta decreased to 18 (95% CI, 16-21, *P* < 0.001) and 8 (95% CI, 6-10, *P* < 0.001) min of CPB and AoX times, respectively.

The length of hospital stay did not differ between groups (6.5 vs 7.0 days, with a difference of 0.4 days [95% CI, 0.1-0.9, *P* = 0.255]).

Binary logistic regression analysis showed that CSA was not associated with postoperative complications: in-hospital mortality (OR= 0.76, 95% CI, 0.43-1.34, *P* = 0.350), in-hospital CVA/TIA (OR= 0.52, 95% CI, 0.26-1.06, *P* = 0.071), in-hospital CVA (OR = 0.50, 95% CI, 0.22-1.18, *P* = 0.116), in-hospital TIA (OR = 0.59, 95% CI, 0.16-2.14, *P* = 0.425), pneumonia (OR = 0.71, 95% CI, 0.47-1.09, *P* = 0.118), urinary tract infection (OR = 1.05, 95% CI, 0.52-2.12, *P* = 0.897), ICU re-admission (OR = 1.16, 95% CI, 0.75-1.77, *P* = 0.506), and renal failure (OR= 1.57, 95% CI, 0.94-2.61, *P* = 0.083). Peri- and postoperative outcomes are displayed in **Tables 2 and 3**.

**Table 2.** Peri- and Postoperative Continuous Outcomes

	Non-CSA group N = 2179	CSA group N = 1081	*P*-value	Delta	Adjusted *P*-value	Delta after adjustment
CPB time (min)	80.4 (SD 45)	111 (SD 50)	<0.001	31	<0.001^a^	18
AoX time (min)	52 (SD30)	67 (SD 33)	<0.001	15	<0.001^a^	8
Length of hospital stay (days)	6.5 (SD 5.3)	6.95 (7.9)	0.419	–	0.255^b^	–

Delta = B coefficient, representing the mean difference in time (min) between the CSA and non-CSA groups.

a *P*-value based on linear regression corrected for age, CPB use, renal impairment, LVEF <30%, PVD, DM, and EuroSCORE II.

b *P*-value based on T-test with log-transformed outcome data plus linear regression corrected for age, CPB use, renal impairment, LVEF <30%, PVD, DM, and EuroSCORE II.

Abbreviations: AoX: aortic cross clamping; CPB: cardiopulmonary bypass; CSA: concomitant surgical ablation; SD: standard deviation.

**Table 3.** Postoperative Outcomes

Postoperative outcomes^a^	OR	95% CI	*P*-value
In-hospital mortality	0.76	0.43-1.34	0.350
In-hospital CVA/TIA	0.52	0.26-1.06	0.071
In-hospital CVA	0.50	0.22-1.18	0.116
In-hospital TIA	0.59	0.16-2.14	0.425
Pneumonia	0.71	0.47-1.09	0.118
Urinary tract infection	1.05	0.52-2.12	0.897
ICU re-admission	1.16	0.75-1.77	0.506
Renal failure	1.57	0.94-2.61	0.083

a Reference is concomitant surgical ablation. All analysis corrected for age, CPB use, renal impairment, LVEF <30%, PVD, DM, and EuroSCORE II. Abbreviations: CI: confidence interval; CVA: cerebrovascular accident; ICU: intensive care unit; OR: odds ratio; TIA: transient ischaemic attack.

### Quality of life

MLM was conducted to evaluate physical and mental QoL. MLM enabled the inclusion of repeated measures and accounted for both fixed and random effects allowing the analysis of 766 patients. In the CSA group (*N* = 242), the mean physical health score improved from 52.5 to 66.4 during follow-up, with a difference of 13.9 points (95% CI, 10.7-17.1 *P* < 0.001), whereas in the non-CSA group (*N* = 524), the score went from 55.2 to 65.8 with a difference of 10.6 points (95% CI, 8.4-12.8, *P* < 0.001). MLM were applied to assess differences of improvement between groups over time, showing no significant difference (*P* = 0.091).

**Figure 2.**
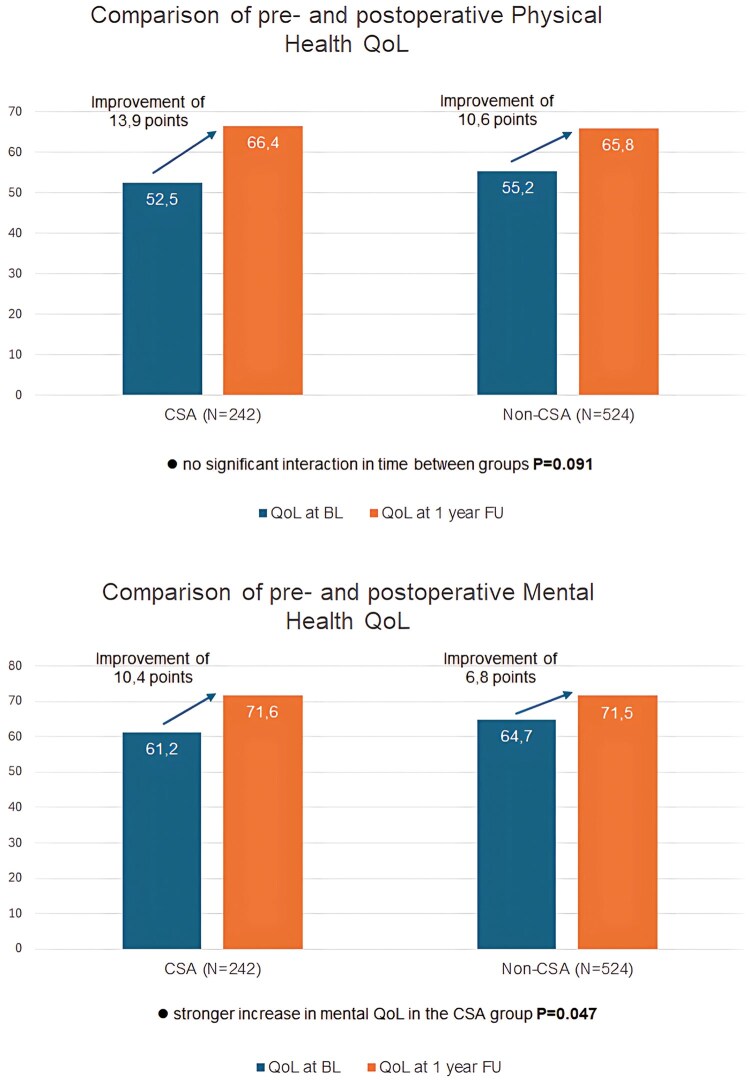
Comparison of Physical and Mental Health QoL Pre- and Postoperative. Abbreviations: BL: base line; CSA: concomitant surgical ablation; FU: follow-up; QoL: quality of life

Mental health scores also improved in the CSA group from 61.2 to 71.6 with a difference of 10.4 points (95% CI, 7.5-13.5, *P* < 0.001) and in the non-CSA group from 64.7 to 71.5 with a difference of 6.8 points (95% CI, 4.7-8.8, *P* < 0.001). MLM showed a significant group by time interaction, indicating a stronger increase in mental QoL in the CSA group (*P* = 0.047) (**Figure 2**).

### Trends of performing CSA

**Figure 3.**
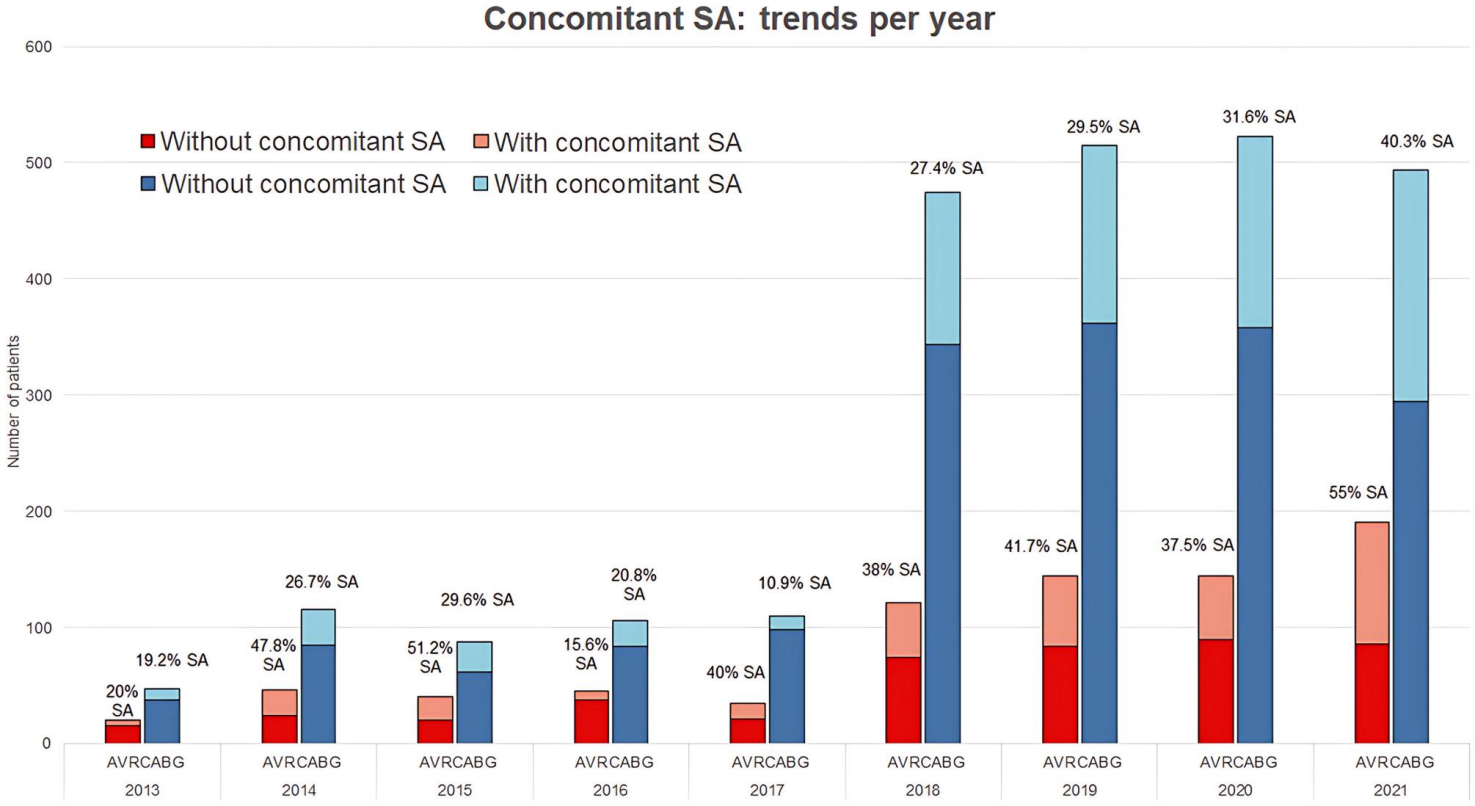
Yearly Overview of CSA Performance, Showing the Percentage of Patients Who Received CSA. A significant increase in registered operations is observed from 2018 on, indicating underreporting of AF as preoperative characteristic and less accurate representation of the surgical trends before this year. Abbreviations: AVR: aortic valve replacement; CABG: coronary artery bypass grafting; SA: surgical ablation

**Figure 3** shows the total volume per year of CABGs or AVRs performed in AF patients. From 2013 to 2017, we observed low proportions of patients operated per year. From 2018 to 2021, an increasing tendency of performing CSA was observed in the mixed population, from 29.7% in 2018 to 44.4% in 2021. The rates increased from 27.4% to 40.3% in CABG patients and from 38.8% to 55.0% in AVR patients.

CSA was more frequently performed in AVR patients compared to CABG patients, with stable proportions, except for a notable increase in 2021 when CSA became more common than non-CSA procedures.

### Subanalysis per intervention group

The survival analysis of the different intervention types (CABG and AVR) showed no statistically significant difference between the CSA and non-CSA groups.

QoL scores improved postoperatively for both CABG and AVR patients, with no significant group by time interactions.

A detailed description of the subanalyses can be found in the **Supplementary Material**.

## DISCUSSION

This study was conducted using a large national database from the NHR and aimed to evaluate the outcomes and safety of performing CSA in AF patients undergoing the most common non-mitral valve surgeries: CABG and AVR. We found that CSA patients presented longer CPB and AoX times, but no significant differences in postoperative complications such as in-hospital mortality, stroke, pneumonia, or renal failure. CSA was associated with improved survival and mental QoL compared to non-CSA.

### Intra- and postoperative comparison

CSA patients had longer operation times. Specifically, the mean CPB-time in the CSA group was 18 min longer, while the AoX-time was 8 min longer than in the non-CSA group. However, these time extensions are clinically unlikely to be relevant, as supported by the findings of this study, which showed no significant differences in major postoperative outcomes, such as in-hospital mortality, stroke, pneumonia, or renal failure. Literature on these specific variables (CPB and AoX times) is limited, as they are often not reported. The PRAGUE-12 trial,^8^ a randomized multicentre study published in 2012, included 224 AF patients undergoing valve and/or coronary surgery, who were randomized to receive CSA or not, revealing that CPB was 28 min longer and AoX, 27 min longer in the CSA group. Although these values differ from our findings, a direct comparison is challenging due to the inclusion of more complex procedures in the PRAGUE-12 trial and its limited patient population. To our knowledge, no recent studies have specifically compared CPB- and AoX times in patients undergoing isolated CABG or AVR with or without CSA. Although in our data no information is available regarding the lesion set applied, this difference observed in operation times could suggest a less complex ablation such as pulmonary vein isolation.

A key finding is that early mortality and in-hospital CVA/TIA rates did not significantly differ between groups. This contrasts with the results of the STS database study,^5^ where CSA was associated with lower 30-day mortality and stroke, but aligns with observations from other studies.^7^^,^^8^ Future research should account for contemporary surgical strategies and consider the role of left atrial appendage (LAA) occlusion, as it may be a confounder for postoperative CVA/TIA, as demonstrated in the LAAOS III trial,^15^ which linked LAA occlusion to a reduced risk of ischaemic stroke.

Another interesting observation in our data was that a higher proportion of off-pump CABG was observed in the non-CSA group. This can suggest that CSA is generally less frequently performed when CPB is not used, maybe due to a more challenging technique or haemodynamic problems when ablating the pulmonary veins. Studies focused on evaluating off-pump CABG with CSA could offer insights into this topic and its potential consequences.

### Follow-up

Regression analyses showed a statistically significant survival difference favouring CSA patients. This finding contributes to the limited and sometimes contradictory evidence regarding the mid- and long-term effects of CSA. However, our results align with a general trend observed by other studies^16–18^ which showed improved survival in CSA patients. The exact reasons for this survival advantages remain unclear, but evidence from randomized control trials (RCT), such as CASTLE-AF^19^ and EAST-AFNET 4,^20^ suggest that a more aggressive rhythm control strategy may reduce mortality and cardiovascular events in AF patients. Although the mechanisms driving these benefits are not yet fully understood, these findings emphasize the importance of future research to further investigate the impact of assertive rhythm control.

We also observed an improvement in QoL in both groups, with stronger effects in the CSA cohort. Improved QoL is often associated with freedom from AF and sinus rhythm maintenance,^21^ potentially related to reduced medication use and fewer hospital visits.

### Trends

This study aimed to illustrate the frequency of CSA performance over the years. Prior to 2018, the number of surgical interventions reported was very limited when compared to 2018 onwards, likely reflecting underreporting of AF in the NHR. Consequently, the percentage of CSA before 2018 may be overestimated and trends from that period are not fully representative. Consequently, analysis of the trends should focus on data from 2018 until 2021. In these recent years, the increase in AF and CSA registration may relate to updated clinical guidelines emphasizing its beneficial effects, as seen in a recently published study from the USA,^22^ showing CSA rates increased from 2.1% to 17.4% after the STS guideline update.^4^

A multicentre study published in 2024 from Poland^11^ reported CSA (with or without LAA occlusion) in 10.26% of AF patients undergoing cardiac surgery, with comparable low rates for isolated CABG (9.6%) and AVR (11.3%). A previous Polish study^9^ found CSA in only 4.4% of AF patients undergoing CABG. In contrast, two STS studies^18^^,^^23^ reported higher CSA rates of 30.5% in AF patients undergoing CABG and 33.1% in AVR patients. In this study, the CSA rate reached 44.4% in the final study year. The rise in CSA procedures may reflect updated guidelines and research highlighting its potential benefits. As surgical strategies vary by country, presenting country-specific data is essential to refine techniques and outcomes.

### Limitations

The NHR database was initially designed for quality-control rather than for research purposes. Therefore, certain key variables, such as AF type, left atrial dimensions, lesion set, energy source, LAA exclusion, or other long-term outcomes, are either missing or insufficiently documented, limiting specific analyses.

Additionally, AF registration before 2018 was incomplete, possibly leading to an overestimation of CSA rates in earlier years, which limits the analysis of trends.

Subanalyses of the AVR and CABG groups showed no significant survival differences within these subgroups, likely due to smaller sample sizes, highlighting the need for larger studies to assess specific intervention groups.

The reasons for not performing CSA remain unclear, with potential selection bias favouring patients with fewer comorbidities, smaller atria, or less advanced types of AF. While PSM adjusts for confounders, it does not replace randomization, so the results should be interpreted with caution.

## CONCLUSION

This nationwide, retrospective multicentre registry based study in AF patients undergoing isolated CABG or AVR found that CSA was associated with improved survival and better postoperative mental QoL. While CSA was associated with slightly longer CPB- and AoX times, it showed no significant differences in major postoperative complications, including mortality, stroke, and renal failure. CSA rates increased from 29.7% in 2018 to 44.4% in 2021, reflecting significant progress and positioning Netherlands ahead internationally. While this is encouraging, there is significant room for further improvement through enhanced care implementation and ongoing research.

## Supplementary Material

ezaf244_Supplementary_Data

## Data Availability

The data that support the findings of this study are available from the NHR upon reasonable request.
