# Supplementary material for: Concomitant Surgical Ablation in Atrial Fibrillation Patients Undergoing Cardiac Surgery for Isolated Coronary and Aortic Valve Disease: A Multicentre Study from The Netherlands Heart Registration
Source: Eur J Cardiothorac Surg. 2025 Jul 28;67(8):ezaf244. doi: 10.1093/ejcts/ezaf244 (PMC12342894; doi:10.1093/ejcts/ezaf244)
Supplement: ezaf244_Supplementary_Data [file ezaf244_supplementary_data.zip › Supplementary Material.docx]

**Supplementary Material**

Postoperative permanent pacemaker implantation within 30 days

Data on postoperative pacemaker implantation within 30 days was only available for 867 patients, with a percentage of missing data of 72.3%. In the CSA group (N= 264) 9 patients (3.4% of the available data) received a permanent pacemaker compared to 18 patients (3.0% of the total available) in the non-CSA group (N=603) (P=0.741). After applying binary logistic regression CSA was not associated to added odds of pacemaker implantation within 30 days (OR= 1.37, 95% CI 0.50-3.74, P=0.544).

Due to the high percentage of missing data, these results were not included in the final manuscript.

Sub-analysis per intervention group

Survival

The survival analysis of the different intervention types (CABG vs AVR) showed no statistically significant difference between the CSA and non-CSA groups after adjusting for confounders.

CABG patients undergoing CSA presented 5-year survival rates of 91.8% and 86% for non-CSA patients (P = 0.156). AVR patients showed 5-year survival rates of 94.0% for CSA patients and 87.6% for non-CSA patients (P = 0.203).

QoL scores

CABG

Mixed linear models was applied, allowing the analysis of 591 CABG patients. Physical health scores in the CSA group (N= 178) improved from 53.4 to 66.0 (P <0.001) whereas in the non-CSA group (N = 413) the improvement was from 54.9 to 65.5 (P <0.001). Time by group (CSA vs non-CSA) interactions showed no significant differences between groups (P=0.872).

Mental health scores increased from 62.6 to 72.1 (P <0.001) in the CSA group (N = 178), and from 64.6 to 71.2 (P <0.001) in the non-CSA group (N = 413). Time by group (CSA vs non-CSA) interactions showed no significant differences between groups (P=0.189).

AVR

Mixed linear models was applied, allowing the analysis of 175 AVR patients. Physical health scores in the CSA group (N =66) improved from 50.1 to 67.1 (P <0.001), while in the no-CSA group (N = 109) scores increased from 56.4 to 67.1 (P <0.001). Time by group (CSA vs non-CSA) interactions showed no significant differences between groups (P=0.095).

Mental health scores in the CSA group (N = 66) group increased from 57.5 to 70.3 (P <0.001) while in the non-CSA group (N = 109) they improved from 65.4 to 72.8 (P <0.001). Time by group (CSA vs non-CSA) interactions showed no statistical significance between groups (P=0.158)

Additional definitions and concepts:

1. Definition for entry of “concomitant ablative surgery for AF” in the Netherland Heart Registration database: “Surgical treatment of (supra)ventricular arrhythmias. This includes surgical ablations such as the Maze procedure (including mini-Maze), PVI (pulmonary vein isolation), cryoablation for ventricular tachycardia, endocardial resections, and others.”

2. Quality of life registration in the NHR database: Quality of life is measured using the SF-36 questionnaire (version 2 or version 1) or the SF-12 questionnaire (version 2). Baseline questionnaires are completed by the patient previous to the operation (with a maximum period of 2 months before surgery). Follow-up questionnaires are coded as “1-year follow-up” and are obtained 10 to 14 months after surgery. The described methods apply for all patients, independently of the type of intervention, performance of CSA and other characteristics.

**Tables and figures**

| Name | Function | Medical Centre |
| --- | --- | --- |
| Dr. S. Bramer | Cardio-thoracic surgeon | Amphia Ziekenhuis |
| Dr. R.A.F. de Lind van Wijngaarden | Cardio-thoracic surgeon | Amsterdam UMC |
| Dhr. B.M.J.A. Koene | Cardio-thoracic surgeon | Catharina Ziekenhuis |
| Dr. J.A. Bekkers | Cardio-thoracic surgeon | Erasmus MC |
| Dr. G.J.F. Hoohenkerk | Cardio-thoracic surgeon | HagaZiekenhuis |
| Dr. A.L.P. Markou | Cardio-thoracic surgeon | Isala |
| Dhr. A. de Weger | Cardio-thoracic surgeon | Leids Universitair Medisch Centrum |
| Dr. P. Segers | Cardio-thoracic surgeon | Maastricht UMC+ |
| Dr. D. Stecher | Cardio-thoracic surgeon | Medisch Centrum Leeuwarden |
| Dr. R.G.H. Speekenbrink | Cardio-thoracic surgeon | Medisch Spectrum Twente |
| Dr. V.G. Hindori | Cardio-thoracic surgeon | Onze Lieve Vrouwe Gasthuis |
| Dhr. W.W.L. Li | Cardio-thoracic surgeon | Radboudumc |
| Dhr. E.J. Daeter | Cardio-thoracic surgeon | St. Antonius Ziekenhuis |
| Dr. M.M. Mokhles | Cardio-thoracic surgeon | UMC Utrecht |
| Dr. Y. Douglas | Cardio-thoracic surgeon | Universitair Medisch Centrum Groningen |

**Supplementary Table 1.** Members of the Cardiothoracic Surgery Registration Committee of the Netherlands Heart Registration.

| Treatment group | Interval (years) | Number of patients at the beginning of the interval | Number of patients withdrawing during interval | Number of Patients Exposed to risk | Number of terminal events | Cumulative proportion surviving at end of interval | Standard error of Cumulative proportion surviving at end of interval | Hazard rate | Standard error of Hazard rate |
| --- | --- | --- | --- | --- | --- | --- | --- | --- | --- |
| Non- CSA | 1 | 2178 | 322 | 2017 | 147 | 0,93 | 0,01 | 0,08 | 0,01 |
|  | 2 | 1709 | 418 | 1500 | 63 | 0,89 | 0,01 | 0,04 | 0,01 |
|  | 3 | 1228 | 422 | 1017 | 43 | 0,85 | 0,01 | 0,04 | 0,01 |
|  | 4 | 763 | 347 | 589,5 | 24 | 0,82 | 0,01 | 0,04 | 0,01 |
|  | 5 | 392 | 232 | 276 | 20 | 0,76 | 0,02 | 0,08 | 0,02 |
| CSA | 1 | 1081 | 288 | 937 | 40 | 0,96 | 0,01 | 0,04 | 0,01 |
|  | 2 | 753 | 216 | 645 | 17 | 0,93 | 0,01 | 0,03 | 0,01 |
|  | 3 | 520 | 190 | 425 | 14 | 0,9 | 0,01 | 0,03 | 0,01 |
|  | 4 | 316 | 140 | 246 | 7 | 0,88 | 0,01 | 0,03 | 0,01 |
|  | 5 | 169 | 116 | 111 | 3 | 0,85 | 0,02 | 0,03 | 0,02 |

**Supplementary Table 2.** Life Table. CSA = concomitant surgical ablation.


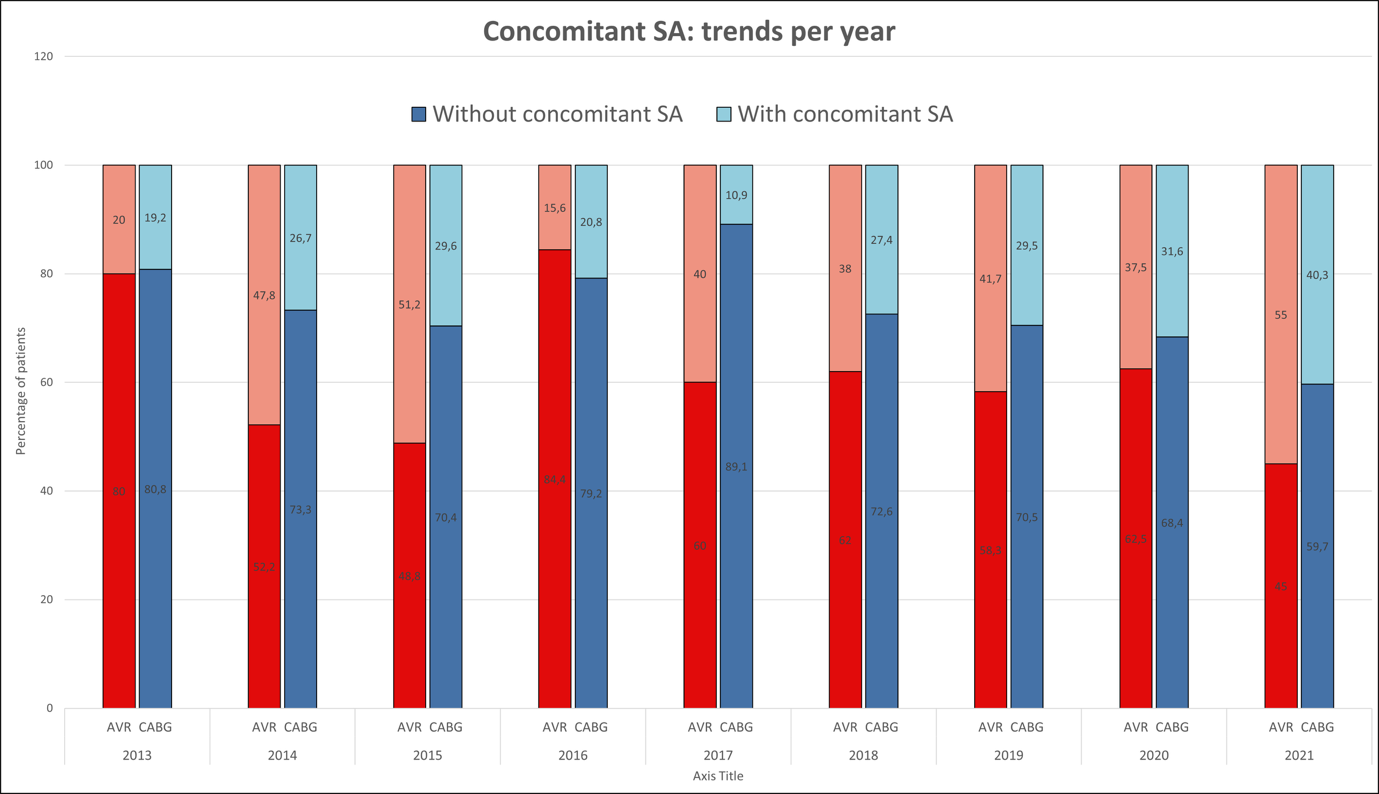


**Supplementary Figure 1.** Yearly overview of CSA performance, showing the percentage of patients who received CSA. AVR = aortic valve replacement, CABG = coronary artery bypass grafting, SA = surgical ablation.
